# Supplementary material for: Information and Communications Technology as a Health Promotion Method for Older Adults in Assisted-Living Facilities: Three-Arm Group-Randomized Trial
Source: JMIR Aging. 2019 May 6;2(1):e12633. doi: 10.2196/12633 (PMC6716436; doi:10.2196/12633)
Supplement: Multimedia Appendix 1 [file aging_v2i1e12633_app1.pdf]

Multimedia Appendix 1. Test of mean changes in quality of life, social support, happiness, and depressive symptoms after a 12-week intervention across the three groups

Outcome	Within-group change, mean±SD									Test for Between-group change [a]	
	ICT- communication group (A, n=19)			ICT-entertainment group (B, n=18)			Usual Care(C, n=17)				
	Pre	Post	Change	Pre	Post	Change	Pre	Post	Change	*P-value*	Post Hoc
HRQOL -Physical Component	27.70 ±18.51	27.24 ±16.61	-0.46	25.83 ±17.06	46.32 ±11.86	20.49***	25.88 ±23.00	24.63 ±23.54	-1.25	.002	B>A**, A>C, B>C
HRQOL -Mental Component	33.88 ±18.14	37.99 ±16.28	4.11*	31.60 ±16.46	68.92 ±15.81	37.32***	36.03 ±27.31	33.33 ±25.04	-2.71	.000	B>A**,A>C , B>C**
Social Support - Family/Friend	1.29 ±0.21	1.34 ±0.19	1.48**	1.34 ±0.29	1.38 ±0.30	0.04***	1.51 ±0.31	1.51 ±0.31	0	.259	A>B,A>C, C>B
Social Support-Healthcare Workers	1.50 ±0.24	1.52 ±0.20	0.02	1.68 ±0.17	1.78 ±0.27	0.1**	1.64 ±0.19	1.61 ±0.18	-0.03	.002	B>A*,A>C, B>C
Social Support-Overall satisfaction	2.16 ±0.31	2.36 ±0.24	0.2***	2.34 ±0.28	2.55 ±0.21	0.21***	2.32 ±0.21	2.34 ±0.16	0.02	.008	B>A,A>C, B>C
Happiness	3.21 ±3.18	4.00 ±4.06	0.79*	4.17 ±4.36	7.89 ±3.53	3.72**	3.47 ±3.89	3.71 ±3.95	0.24	.033	B>A,A>C, B>C
Depressive Symptom Scale	13.74 ±4.63	11.00 ±3.27	-2.74**	13.11 ±4.63	5.78 ±3.27	-7.33***	11.06 ±4.08	11.76 ±4.64	0.7	.000	A>B**,C>A ,C>B**

a: P-value within-group change: *<.05 **<.01 ***<.001
b: P-value across three groups were compared using the Kruskal-Wallis Test and Dunn's nonparametric comparison for post hoc KW testing: *<.005 **<.001 were discerned significant after the Bonferroni adjustment.
