# Supplementary material for: Information and Communications Technology as a Health Promotion Method for Older Adults in Assisted-Living Facilities: Three-Arm Group-Randomized Trial
Source: JMIR Aging. 2019 May 6;2(1):e12633. doi: 10.2196/12633 (PMC6716436; doi:10.2196/12633)
Supplement: Multimedia Appendix 2 [file aging_v2i1e12633_fig.pdf]

Original Paper

Information and Communications Technology as a Health Promotion Method for Older Adults in Assisted-Living Facilities: Three-Arm Group-Randomized Trial

Ching-Ju Chiu, PhD; Chi Hui Wu, RN, MS

Institute of Gerontology, College of Medicine, National Cheng Kung University, Tainan, Taiwan

Corresponding Author:
Ching-Ju Chiu, PhD
Institute of Gerontology
College of Medicine
National Cheng Kung University
No. 1 University Road
Tainan,
Taiwan
Phone: 886 6 2353535 ext 5739
Fax: 886 6 3028175
Email: cjchiu@mail.ncku.edu.tw

Abstract

Background: The application of technology is an important and growing aspect in the field of long-term care. Growing evidence shows the positive impact of technology aids in helping the lives of the elderly. However, it is not known which aspects of information and communications technology (ICT) are preferred by older adults living in long-term care facilities.

Objective: The goal of the research was to compare the impact of ICT-communication, ICT-entertainment, and conventional care on older adults' health and psychological change after interventions among older adults in assisted-living facilities.

Methods: A three-arm group-randomized trial design was used to evaluate participants who resided in three different but comparable assisted-living facilities and received different aspects of the ICT interventions. A total of 54 older adults with disabilities received one of the three interventions over 12 weeks and completed pre- and postevaluations on quality of life, social support, and psychological well-being.

Results: Participants completing this study had a mean age of 73 (SD 11.4) years, and 50% (27/54) were male. Both the ICT-communication and ICT-entertainment groups showed significant improvement in the mental component of quality of life (4.11, P=.012 and 37.32, P<.001, respectively), family/friend–related social support (0.05, P=.001 and 0.04, P<.001, respectively), happiness (0.79, P=.038 and 3.72, P=.001, respectively), and depressive symptoms (–2.74, P=.001 and –7.33, P<.001, respectively). Importantly, participants in the ICT-entertainment group improved significantly more than the other two groups. The ICT-entertainment group also showed improvement in the physical component of quality of life (20.49, P<.001) and health care worker–related social support (0.1, P=.008).

Conclusions: Results suggest that the entertainment but not the communication part of ICT is the most effective health promotion method for improving the health and psychological well-being of older adults in assisted-living facilities.

(JMIR Aging 2019;2(1):e12633) doi:10.2196/12633

KEYWORDS

information and computer technology; quality of life; social support; psychological well-being; long-term care facilities; disabled; elderly; disability; assisted living; seniors; geriatrics

Introduction

Information and communications technology (ICT) apps have become foundational in aging communities worldwide. The internet is transforming the way older adults communicate and socialize with family and friends [1]. Particularly in older adults, social networking is becoming a general contributor to this communication, reflecting better overall health [2], and new possibilities are opening up through the concept of social capital and its potential relevance as a theoretical tool for investigating

XSL•FO
RenderX

aging [3]. However, disabilities can affect psychological health along with the subjective sense of well-being [4], particularly among institutional care residents who are facing social barriers [5]. Social barriers are related to the social determinants of health, the conditions in which people are born, grow, live, learn, work, and age [6], causing people with these barriers to be less likely to mingle with or get support from family and friends, increasing disassociation with society. These barriers can result in a sense of negative well-being that may include feelings of abandonment or being left behind, affecting older people's outlook on life and in turn their quality of life [7].

Interventions involving the use of assistive technology may help overcome social barriers for older adults who live in long-term care facilities and thus improve their quality of life and sense of well-being [8,9]. However, there have been limited studies evaluating the efficacy of the use of touch screen devices in older adults who live in long-term care facilities, and research comparing communication and entertainment apps and their impact on the health and well-being of this population is lacking.

The objective of this study was to evaluate the impact of ICT on psychological well-being, quality of life, and physical and cognitive functioning in older adults living in long-term care facilities. In addition, we compared the effectiveness of ICT between those receiving the entertainment aspect versus those receiving the communication aspect of ICT learning to ascertain which aspect of the ICT was more welcomed by the population.

Methods

Research Design and Participants

A group-randomized trial design was employed in the study, for which three comparable community-based long-term care facilities in Southern Taiwan were selected. This study was approved by the institutional review board of National Cheng Kung University Hospital in Taiwan (number A-ER-102-425). No changes were made to the trial design after pretrial approval from the review board.

A total of 286 adults were living in the three facilities. After excluding those aged younger than 50 years, those unable to comply, and those who exhibited confusion or agitation, the remaining 86 adults, including 28 in site A, 32 in site B, and 26 in site C, were invited to participate. Each site was randomized to receive one of the three intervention methods: the ICT-communication (using Line, a freeware app for instant communications on electronic devices) group, ICT-entertainment (using YouTube, a video sharing website) group, and usual care group.

Only 66% (57/86) of participants signed informed consents, consisting of 19 in each group. Among them, 3 participants dropped out during the intervention (one moved to another city and the other two passed away), which resulted in a total of 54 participants (retention rate 95%) comprising the ICT-communication group (19/54, 35%), ICT-entertainment group (18/54, 33%), and usual care group (17/56, 30%). The research period from commencement to completion was May to September 2015.

Intervention Process

The usual care group included observation participants with routine daily activities. Participants allocated to the ICT-communication and ICT-entertainment groups received ICT training once a week for 12 weeks (see Table 1). The author and a research assistant led each ICT session and worked with no more than 10 participants in a group. Each session lasted about 90 minutes, with visual demonstrations using an overhead projector and hands-on exercises on all the contents. Touch screen tablets with individualized assistance devices attached, such as tablet holders and sensor pens especially designed for a specific disability, were used as the technology devices. Each class included a demonstration of the new skill and a repetition drill for learning. Each class also included a review and practice of the skill learned in the previous class.

In addition to training on general knowledge related to the tablet functions and inputting of data, the ICT-communication group participants learned how to invite friends to join in using the Line app, make a video call, download a sticker, send a photo card, send a text message, and attach a file through Line and send it to friends. Participants in the ICT-entertainment group learned how to search on YouTube for their favorite songs, TV shows, and videos.

Measures

All participants were interviewed in person and completed questionnaires at pre- and postintervention. The questionnaires include questions on sociodemographic variables, health-related quality of life (12-Item Short Form Health Survey [SF-12]), Taiwanese Inventory of Social Supportive Behavior (TISSB), Chinese Person's Happiness Inventory (CHI), and Center for Epidemiologic Studies Depression Scale (CES-D).

Cognitive Function

Cognitive ability assessments were conducted using a structured measure of cognitive performance, the Short Portable Mental Status Questionnaire (SPMSQ), a mechanism with high validity and test-retest reliability [10]. This 10-item structured survey is a widely used questionnaire designed to measure several intellectual areas including orientation, short- and long-term memory, general knowledge, and deciphering issues. The Chinese version of the SPMSQ has been authenticated in earlier studies [11], and a higher rating indicates a better cognitive intellectual capacity score. Cronbach alpha in our sample was .60 [12].

XSL•FO
RenderX

Table 1. Comparison of the curricula for the two intervention groups.

Class	Syllabus	
	ICT[a]-communication group	ICT-entertainment group
1	General knowledge about tablet functions: turn tablet on and off; keyboard functions	General knowledge about tablet functions: turn tablet on and off; keyboard functions
2	Input data using handwriting, typing, and voice commands for searching	Input data using handwriting, typing, and voice commands for searching
3	Invite friends to join; take and attach photos; add friends by shaking or using Quick Response feature	Search on YouTube for a movie, news, TV program, or favorite song
4	Make a video phone call using Line	Search on YouTube for a song and sing along (Karaoke)
5	Download a sticker using Line to make a photo card	Search on YouTube for a song or video to express your feelings
6	Make and send a photo card using Line; add sticker graphics	Search on YouTube for a popular song or video about your favorite reminiscence
7	Send a text message using Line and add a sticker	Search on YouTube for a movie, news, TV program, or favorite song and share with your friends
8	Attach a file	Search on YouTube for a song or video that gives you strength when you are having a difficult time
9	Search for a city or street using Google Maps; sightsee using Google Satellite	Search for a city or street using Google Maps; sightsee using Google Satellite
10	Experience YouTube by learning how to search for a favorite song	Experience Line by learning how to make a video or phone call
11	Make a video phone call using Line; send a text message and a sticker	Search on YouTube for a favorite song and sing it with a friend
12	Graduation	Graduation

[a]ICT: information and communications technology.

Physical Functional Status

Functional status was defined as the capacity to perform the activities of daily living (ADL) and the instrumental activities of daily living (IADL). ADL limitations refer to the requirements for accomplishing these 10 basic activities: bathing, dressing, transferring (3 items), going to the lavatory (3 items), and feeding oneself (2 items). Each item ranges from 0 to 5 or 0 to 15, ranging from no difficulty to being totally dependent on others. Total ADL score ranged from 0 to 100, with higher value indicating more disabilities. The IADL assessment included 8 items rating difficulties with participants' ability to go outside, use a telephone, shop, cook, engage in household cleaning, do laundry, take medication, and manage household finances, where each item ranged from 0 to 2 or 0 to 4, representing no difficulty to being totally dependent. Total IADL ranged from 0 to 24, with a higher value indicating a greater degree of disability. Significantly elevated correlations (tau=.83, r=.89, P<.001) were found between this measurement and the assessment of functional status by quality professionals, suggesting good criterion validity. In addition, good interrater reliability has previously been reported for the IADL [13] and the ADL [14].

Depressive Symptoms Scale

The CES-D, developed by Radloff [15], has been widely used in many community-based studies on the elderly with good reliability and validity [16]. The Chinese version of the CES-D scale is a reliable screening instrument for symptoms of depression in the elderly [17] and had good internal consistency (Cronbach alpha=.89) [18]. A total of 11 items were used to ask the participants their feelings over the previous week addressing appetite, level of depression, or degree to which they felt happy or lonely or found people to be unfriendly or to dislike them, were enjoying life, were feeling sad or that life was an effort, were unable to get going, and were experiencing restless sleep (positive items were reverse-scored). The questions had ratings from not at all (0) to always (2). The maximum score was 22, with a higher value indicating more depressive symptoms.

Health-Related Quality of Life

The SF-12, Taiwan version [19], was used in the study to assess health-related quality of life. Its internal consistency and test-retest reliabilities have been shown to be good (ranging from .67 to .82) [20]. There are two factors, interpreted as physical and mental components of health status, in this scale [21]. Each component ranges from 0 to 100. A greater score implies better health-related quality of life.

Social Support Scale

The inventory of the TISSB was used to evaluate participants' social health status [22]. The TISSB version was adapted from the original ISSB scale [23], which included 10 items evaluating emotional support; social integration; informational support; instrumental support from families, friends, and health care workers; and the overall satisfaction with these listed support objects. Each domain of the TISSB was rated from not at all (1) to always (3), with total score ranging from 10 to 30. Scores from 10 to 16 were considered low, 17 to 23 were considered to be moderate, and those 24 and higher were considered to be a high indicator of social support or satisfaction. The TISSB

XSL•FO
RenderX

has been determined to have significant reliability (Cronbach alpha=.89) and validity in older adults in community dwellings [24].

Happiness

Well-being rankings were measured by means of the 10-item CHI [25]. Psychometric analyses revealed good validity and reliability (Cronbach alpha=.95). This project included the happiness measurement scale assessing the well-being of the respondents, including a positive effect, negative effect, and overall satisfaction toward life in the past three months, with each item ranging from 0 to 3. Total scores ranged from 0 to 30, with a greater score representing a higher well-being status.

Statistical Analyses

Data analysis included descriptive and inferential statistical analyses. Changes in the means and standard deviations of the variables at pre- and postinterventions were examined using the Wilcoxon signed-rank test. Intergroup comparisons of changes in the means and standard deviations in the three groups were conducted using the Kruskal-Wallis test and Dunn nonparametric comparison for post hoc Kruskal-Wallis testing. To adjust for multiple comparisons, significance was discerned after the Bonferroni adjustment by setting the alpha levels at <.005 and <.001.

Results

Participant Characteristics

The baseline sociodemographic and health status of the participants is shown in Table 2. Participants had a mean age of 73 (SD 11.4) years; 50% (27/54) were male; 70% (38/54) had less than an elementary school education; 54% (29/54) had an average financial status; average length of stay in long-term care was 36.2 months; average ADL score was 40.2 (higher level of dependence); average IADL score was 6.6 (subtle disabilities); average cognitive score was 9.6 (intact); the majority of the residents (47/54, 87%) had not had previous computer learning experience; and chronic disease count average was 2.3. Participants in the three intervention groups were not statistically significantly different in regard to any of the sociodemographic variables nor were they different in terms of disease, functional status, and variables related to quality of life, depressive symptoms, happiness, social support, and computer learning experience.

Comparison of the Intervention Effect Across the Three Groups

As shown in Multimedia Appendix 1, participants in both the ICT-communication and ICT-entertainment groups statistically significantly increased their quality of life mental component, family and friend social support, satisfaction with support, and happiness and experienced decreases in their depressive symptoms after the 12-week ICT intervention. In addition, participants in the ICT-entertainment group also statistically significantly increased their quality of life physical component and their health care worker–related social support scores after the 12-week intervention.

The results of the Kruskal-Wallis tests comparing changes in the three groups further indicated that the effects of ICT-entertainment and ICT-communication on improving the degree of social support from family and friends and the happiness of older adults living in long-term care facilities did not differ between the ICT-entertainment and ICT-communication groups. The ICT-entertainment group had a significantly greater increase compared to the ICT-communication group in physical and mental component for health-related quality of life and health care worker–related social support and had a greater decrease in depressive symptoms.

XSL•FO
RenderX

Table 2. Baseline characteristics of participants in the ICT-communication group, ICT-entertainment group, and usual care groups.

Characteristics	Total (N=54)	ICT[a]-communication group (n=19)	ICT-entertainment group (n=18)	Usual care group (n=17)	*P* value
**Sociodemography**					
Age (years), mean (SD)	73.0 (11.4)	72.9 (12.3)	72.9 (10.6)	73.2 (11.8)	>.99
Sex (male), n (%)	27 (50)	11 (58)	9 (50)	7 (41)	.61
**Education, n (%)**					.05
Illiterate (yes)	15 (28)	6 (32)	3 (17)	6 (35)	
Elementary school	23 (43)	5 (26)	10 (56)	8 (47)	
High school and above	16 (30)	8 (42)	5 (28)	3 (18)	
**Financial status, n (%)**					.85
Poor	24 (44)	9 (47)	8 (44)	7 (41)	
Average	29 (54)	10 (53)	10 (56)	9 (53)	
Rich	1 (2)	0 (0)	0 (0)	1 (6)	
**Computer learning experience, n (%)**					.39
No	47 (87)	15 (79)	16 (90)	16 (94)	
Yes	7 (13)	4 (21)	2 (11)	1 (6)	
**Health status, mean (SD)**					
LOR[b] (months)	36.2 (37.4)	28.5 (16.9)	50.3 (51.6)	29.3 (33.9)	.39
ADL[c] (0-100)	40.2 (31.5)	31.8 (31.2)	39.2 (29.6)	51.6 (32.5)	.12
IADL[d] (0-24)	6.6 (2.0)	5.8 (2.1)	7.2 (1.9)	6.7 (1.9)	.05
SPMSQ[e] (0-10)	9.6 (0.8)	9.5 (0.8)	9.4 (0.9)	9.7 (0.6)	.56
Disease[f] (count)	2.3 (1.1)	2.2 (1.0)	2.5 (1.3)	2.2 (1.1)	.61
HRQoL[g]–physical component, mean (SD)	26.5 (19.2)	27.7 (18.5)	25.8 (17.1)	25.9 (23.0)	.83
HRQoL–mental component, mean (SD)	33.8 (20.7)	33.9 (18.1)	31.6 (16.5)	36.0 (27.3)	.97
Social support–family/friends, mean (SD)	1.4 (0.3)	1.3 (0.2)	1.3 (0.3)	1.5 (0.3)	.10
Social support–health care workers, mean (SD)	1.6 (0.2)	1.5 (0.2)	1.7 (0.2)	1.6 (0.2)	.06
Satisfaction with social support, mean (SD)	2.3 (0.3)	2.2 (0.3)	2.3 (0.3)	2.3 (0.2)	.09
Happiness, mean (SD)	3.6 (4.0)	3.2 (3.2)	4.2 (4.4)	3.5 (3.9)	.88
Depressive symptom scale, mean (SD)	12.7 (4.4)	13.7 (4.6)	13.1 (4.6)	11.1 (4.1)	.15

[a]ICT: information and communications technology.
[b]LOR: length of residency.
[c]ADL: activities of daily living.
[d]IADL: instrumental activities of daily living.
[e]SPMSQ: Short Portable Mental Status Questionnaire.
[f]Disease count includes diabetes, hypertension, heart disease, cerebral vascular incidents, and others.
[g]HRQoL: health-related quality of life.

Discussion

Principal Findings

The objective of this study was to evaluate the extent to which social support, psychological well-being, and quality of life changed for participants receiving the communication aspect of ICT learning and for participants who received the entertainment aspect of the ICT learning versus the control group with usual care. This study indicates that the entertainment group of ICT learning had the highest change in terms of improving both the physical and mental components of health-related quality of life and happiness and decreasing depressive symptoms as compared with those who were in the communication group of ICT intervention and the usual care group. The ICT-entertainment group also exhibited a significant increase in social support related to health care workers and overall satisfaction with social support.

XSL•FO
RenderX

Previous studies have shown a trend in the communication aspects of ICT using email indicating decreased loneliness and isolation with the potential of improving psychological well-being [8,9,26,27]. Significant differences were found between the pre- and postintervention communication aspects of ICT-communicatiom in terms of the levels of health care worker–related social support and satisfaction with social support. In a prior study by Tsai et al [28], video conferencing with MSN/Skype was used as an intervention for nursing home residents and their families in which the outcome revealed significant changes in levels of social support and loneliness along with decreases in the level of social isolation. However, in our study, typing and texting were both challenging for most of the long-term care facility residents due to physical stiffness, arthritis, and shaky hands, causing an issue with trying to hold a computer tablet or use a touch screen. Residents were given tailored technology accessories such as tablet holders and touch screen pens that helped them overcome these barriers. The software app used in this study was Line; the features of this app involve video phone calling along with texting and the ability to produce and send video and picture files. Additionally, the Line app adds some variety, such as daily free cartoons or emojis that can be attached to messages or videos to communicate with their families or friends.

Interestingly, we found the entertainment aspect of ICT using YouTube was more welcomed by older adults living in long-term care facilities than the communication aspect of ICT using Line. Although participants in both the ICT-communication and ICT-entertainment groups statistically significantly improved in terms of their mental component of quality of life; family and friend social support; satisfaction with the degree of support, happiness, and depressive symptoms; participants in the ICT-entertainment group revealed a statistically significantly greater mental component score improvement and a unique improvement on the physical component of the health-related quality of life score. Specifically, while participants in the ICT-communication group improved the mental component of the health-related quality of life score by 4.11 points, the ICT-entertainment group increased by 37.32 points, which is more than two standard deviation increments. Similarly, while there was no significant improvement on the physical component of the health-related quality of life score for participants who received the ICT-communication intervention, participants in the ICT-entertainment group reported statistically significant improvement on the physical component of the health-related quality of life score by increasing 20.49 points, a more than one standard deviation improvement. Post hoc tests reveal statistically significant differences between the two groups, but we believe there is also clinical significance by using ICT-entertainment to improve both the physical and mental components of quality of life than by using the ICT-communication approach for older adults living in long-term care facilities.

ICT-entertainment has greater diversity as an app in terms of meeting the needs of the residents compared with ICT-communication, especially during the ICT learning classes. In addition, YouTube makes it easier to search for songs, movies, news, geography, on-line classes, or something to their interest. This app is a very useful, convenient tool for groups or for use as an individualized entertainment feature to stimulate and motivate the interest of elderly residents, and in addition, makes it possible for them to reminisce about past memories. For example, the YouTube music library options help trigger memories of the past through songs from their youth and family years, helping them to reminisce with family and friends. The residents sang along with music clips and read the text on the screen aloud. Nonverbal communications included clapping their hands and nodding their heads along with the music, dancing, waving their arms in the air, maintaining observation of the screen, observing persons speaking, and maintaining eye contact [29]. Using today's interactive touch screen computer devices is wonderful for older adults. Positive results from multimedia technology increased interaction particularly in the case of songs from their earlier years because it provided a greater depth and variety of materials from their past [30].

It was found that in contrast to the communication aspect of the ICT, which did not show significant improvement in participants' physical quality of life component, the recreation aspects of ICT using YouTube significantly improved participants' physical component of health-related quality of life. This finding echoes those of previous studies indicating that the YouTube app is a feasible means by which to conduct computer-based interactions intended to increase the sense of well-being and improve mood in viewers in order to generate greater communicative participation and engagement in a group [29]. In our study, old songs were found through YouTube for remembrance, comfort, and encouragement that caused the residents to talk and tell stories of their younger years with big smiles on their faces, helping them overcome their negative emotions and frustration. Psychologically, music has been shown to play an important role in emotional self-regulation, communication, and social interaction throughout life and also during the aging process [31]. Both singing and listening to music improve mood, orientation, and remote episodic memory and, to a lesser extent, attention, executive functions, and general cognition. Singing also enhances short-term and working memory, whereas music listening has a positive effect on quality of life. Because of advancements in technology, the modern vision for reminiscence has expanded beyond the tangible and traditional [32]. Findings on the effects of YouTube included improved well-being and mood; improved communication, interaction, and engagement; improved quality of life in an institutional care setting; and connection with YouTube reminiscence mediums.

Limitations

This study has some limitations. The sample size was a small convenience sampling of three long-term care facilities. The average participant reported low educational attainment, frailty, and physical disabilities that might have influenced their ability and willingness to achieve training on internet operational procedures or the writing requirements for computer information technology such as text messaging, email, and blogging, among others. We also excluded those who were bedridden or aged younger than 50 years.

XSL•FO
RenderX

Conclusion

Despite these limitations, this study provided evidence that touch screens, lightweight smart computer tablets, and easy-to-use software apps may be a promising form of health promotion activity for older adults living in long-term care facilities even when they are very frail. These leading technologies enable health care workers to assist residents so they can interact with families and friends, including repeat visitors hosting activities or social activities with other residents. In addition, ICT-entertainment has a greater impact in terms of meeting the needs of the residents compared with ICT-communication: it could be used as a sophisticated icebreaker tool to interact with residents, providing excitement and opening up a new means of communication for those who cannot express themselves in the way they desire.

Acknowledgments

This work was supported by a grant from the Taiwan National Science Council (MOST-103-2314-B-006-038-MY3).

Conflicts of Interest

None declared.

Multimedia Appendix 1

Test of mean changes in quality of life, social support, happiness, and depressive symptoms after a 12-week intervention across the three groups.

[PDF File (Adobe PDF File), 181KB - aging_v2i1e12633_app1.pdf]

References

1. Coget J, Yamauchi Y, Suman M. The internet, social networks, and loneliness. It Soc 2002;1(1):180-201 [FREE Full text]
2. Russell C, Campbell A, Hughes I. Ageing, social capital and the Internet: findings from an exploratory study of Australian "silver surfers". Australas J Ageing 2008 Jun;27(2):78-82. [doi: 10.1111/j.1741-6612.2008.00284.x] [Medline: 18713197]
3. Clark E, McCann T. Social capital: one source of wellness in older adults? Health Sociol Rev 2014 Dec 17;12(2):163-170. [doi: 10.5172/hesr.12.2.163]
4. Ku P, McKenna J, Fox K. Dimensions of subjective well-being and effects of physical activity in Chinese older adults. J Ageing 2007;15(4):382-397. [Medline: 18048943]
5. Mitchell J, Kemp B. Quality of life in assisted living homes a multidimensional analysis. J Gerontology B Psychol Sci Soc Sci 2000;55(2):P117-P127. [Medline: 10794190]
6. Centers for Disease Control and Prevention. 2019. Common barriers to participation experienced by people with disabilities URL: https://www.who.int/disabilities/Disability_infographic__EN_rev_Jun_2018.pdf [accessed 2019-03-30] [WebCite Cache ID 77H4Iv1IF]
7. Blaschke CM, Freddolino PP, Mullen EE. Ageing and technology: a review of the research literature. Brit J Soc Work 2009 Mar 20;39(4):641-656. [doi: 10.1093/bjsw/bcp025]
8. Bradley N, Poppen W. Assistive technology, computers and Internet may decrease sense of isolation for homebound elderly and disabled persons. Technol Disability 2003;15(1):19-25.
9. Berkowsky R, Cotton S, Yost E, Winstead V. Attitudes towards and limitations to ICT use in assisted and independent living communities: findings from a specially-designed technological intervention. Educ Gerontol 2013;39(11):797-811. [Medline: 24244065]
10. Pfeiffer E. A short portable mental status questionnaire for the assessment of organic brain deficit in elderly patients. J Am Geriatr Soc 1975 Oct;23(10):433-441. [Medline: 1159263]
11. Chi I, Boey KW. Hong Kong validation of measuring instruments of mental health status of the elderly. Clin Gerontol 1993 Dec 22;13(4):35-51. [doi: 10.1300/J018v13n04_04]
12. Chou K, Chi I. Prevalence and correlates of depression in Chinese oldest old. Int J Geriatr Psychiatr 2005;20(1):41-50. [Medline: 15578666]
13. George L, Fillenbaum G. OARS methodology. J Am Geriatr Soc 1985;33(9):607-615. [Medline: 4031339]
14. Guindon S, Cappeliez P. Contributions of psychological well-being and social support to an integrative model of subjective health in later adulthood. Ageing Int 2010 Jan 14;35(1):38-60. [doi: 10.1007/s12126-009-9050-7]
15. Radloff LS. The CES-D Scale: a self-report depression scale for research in the general population. App Psychol Meas 1977 Jun 01;1(3):385-401. [doi: 10.1177/014662167700100306]
16. Beekman AT, Deeg DJ, Van Limbeek J, Braam AW, De Vries MZ, Van Tilburg W. Criterion validity of the Center for Epidemiologic Studies Depression scale (CES-D): results from a community-based sample of older subjects in The Netherlands. Psychol Med 1997 Jan;27(1):231-235. [Medline: 9122304]
17. Boey K. Cross-validation of a short form of the CES-D in Chinese elderly. Int J Geriatr Psychiatr 1999;14(8):608-617. [Medline: 10489651]

XSL•FO
RenderX

18. Lin C, Su T, Chang M. Quality of sleep and its associ[illegible] factors in the institutionalized elderly. Formosan J Med 2003;7(2):174-184. [doi :10.6320/FJM.2003.7(2).03]
19. Gandek B, Ware J. SF-36 Health Survey: Manual and Interpretation Guide. Boston: Health Institute; 1993.
20. Lam ETP, Lam CLK, Fong DYT, Huang WW. Is the SF-12 version 2 Health Survey a valid and equivalent substitute for the SF-36 version 2 Health Survey for the Chinese? J Eval Clin Pract 2013 Feb;19(1):200-208. [doi: 10.1111/j.1365-2753.2011.01800.x] [Medline: 22128754]
21. Maruish M. User's Manual for the SF-12V2 Health Survey. Lincoln: QualityMetric Incorporated; 2012.
22. Yeh CL. The Correlation Between Social Suport, Health and Depression of Elderly Living in a Self-Paid Institution [master's thesis]. Taichung: Chung Shan Medical College; 1998.
23. Barrera M, Sandler I, Ramsay T. Preliminary development of a scale of social supporttudies on college students. Am J Comm Psychol 1981;9(4):435-447. [doi: 10.1007/BF00918174]
24. Hsu YZ. Correlation Study of the Attitudes Toward Old Age, Social Support, Self-Reported Health Condition and Depression of the Age Between 65- to 74-Year-Old Adults at Lukang Community [master's thesis]. Taipei: Graduate Institute of Health Education, National Taiwan Normal University; 1993.
25. Argyle M, Lu L. The happiness of extraverts. Personality Individ Diff 1990 Jan;11(10):1011-1017. [doi: 10.1016/0191-8869(90)90128-E]
26. White H, McConnell E, Clipp E, Branch LG, Sloane R, Pieper C, et al. A randomized controlled trial of the psychosocial impact of providing internet training and access to older adults. Aging Ment Health 2002 Aug;6(3):213-221. [doi: 10.1080/13607860220142422] [Medline: 12217089]
27. Cotten SR, Anderson WA, McCullough BM. Impact of internet use on loneliness and contact with others among older adults: cross-sectional analysis. J Med Internet Res 2013;15(2):e39 [FREE Full text] [doi: 10.2196/jmir.2306] [Medline: 23448864]
28. Tsai H, Tsai Y, Wang H, Chang Y, Chu HH. Videoconference program enhances social support, loneliness, and depressive status of elderly nursing home residents. Aging Ment Health 2010 Nov;14(8):947-954. [doi: 10.1080/13607863.2010.501057] [Medline: 21069600]
29. O'Rourke J, Tobin F, O'Callaghan S, Sowman R, Collins D. YouTube: a useful tool for reminiscence therapy in dementia? Age Ageing 2011;40(6):742-744. [Medline: 21880626]
30. Harley D, Fitzpatrick G. YouTube and intergenerational communication: the case of Geriatric1927. Univ Access Inf Soc 2008 May 29;8(1):5-20. [doi: 10.1007/s10209-008-0127-y]
31. Juslin P, Sloboda J. Handbook of Music and Emotion: Theory, Research, Applications. Oxford: Oxford University Press; 2011.
32. Astell A. Facilitating communication in dementia with multimedia technology. Brain Lang 2004 Oct;91(1):80-81. [doi: 10.1016/j.bandl.2004.06.043]

Abbreviations

ADL: activities of daily living
CES-D: Center for Epidemiologic Studies Depression Scale
CHI: Chinese Person’s Happiness Inventory
IADL: instrumental activities of daily living
ICT: information and communications technology
TISSB: Taiwanese Inventory of Social Supportive Behavior
SF-12: 12-Item Short Form Health Survey
SPMSQ: Short Portable Mental Status Questionnaire

Edited by G Eysenbach; submitted 30.10.18; peer-reviewed by V Manchaiah, J Gaugler; comments to author 20.12.18; revised version received 18.02.19; accepted 24.03.19; published 19.04.19

Please cite as:
Chiu CJ, Wu CH
Information and Communications Technology as a Health Promotion Method for Older Adults in Assisted-Living Facilities: Three-Arm Group-Randomized Trial
JMIR Aging 2019;2(1):e12633
URL: http://aging.jmir.org/2019/1/e12633/
doi:10.2196/12633
PMID:

XSL•FO
RenderX

©Ching-Ju Chiu, Chi Hui Wu. Originally published in JMIR Aging (http://aging.jmir.org), 19.04.2019. This is an open-access article distributed under the terms of the Creative Commons Attribution License (https://creativecommons.org/licenses/by/4.0/), which permits unrestricted use, distribution, and reproduction in any medium, provided the original work, first published in JMIR Aging, is properly cited. The complete bibliographic information, a link to the original publication on http://aging.jmir.org, as well as this copyright and license information must be included.

XSL•FO
RenderX
